# Layer-Inversion Zones in Angular Distributions of Luminescence and Absorption Properties in Biaxial Crystals

**DOI:** 10.3390/ma3042474

**Published:** 2010-03-31

**Authors:** Yannick Petit, Patricia Segonds, Simon Joly, Benoît Boulanger

**Affiliations:** 1 Institut Néel CNRS/UJF, 25 rue des Martyrs, BP 166, F38402 Grenoble Cedex 9 France; E-Mails: patricia.segonds@grenoble.cnrs.fr (P.S.); simon.joly@grenoble.cnrs.fr (S.J.); benoit.boulanger@grenoble.cnrs.fr (B.B.); 2 Université de Genève, 20 rue de l’Ecole de Médecine, CH-1211 Genève 4, Switzerland

**Keywords:** crystal optics, biaxial crystals, linear spectroscopy, luminescent materials

## Abstract

We numerically depict the complete angular distributions of luminescence and absorption properties in biaxial media, by calculating the imaginary part of the optical index for all directions of propagation. Our simulations show a double-layer surface with specific topology and symmetry properties that greatly differ from those of the refractive index surface. Our calculations show that the two layers intersect and inverse themselves along continuous loci related to polarization-independent luminescence or absorption properties. Specificities related to the orthorhombic, monoclinic and triclinic biaxial crystal systems are discussed. Such theoretical developments should be considered to fully exploit innovating luminescent materials.

## 1. Introduction

On-going progress in crystal growth provides new crystals with potential luminescent properties when doped with Rare-Earth ions [1]. It is remarkable that most of such new materials are low-symmetry media, belonging to the orthorhombic or monoclinic biaxial crystal systems. The theoretical frame of linear spectroscopy is fulfilled in crystals belonging to the isotropic and uniaxial optical classes [2]. However, it is not the case for biaxial crystals, even if some theoretical results have been already reported in orthorhombic or monoclinic media [2,3,4]. Therefore, it is still challenging to develop a well-defined theoretical frame, so as to provide a complete description of luminescence and absorption properties in biaxial media. Such theoretical developments are today a necessity to properly characterize and then to fully exploit the applicative potential of new materials for optics. The present paperis thus directly motivated by the new issues brought by the synthesis of new biaxial materials with innovative luminescence properties [3,4,5].

Linear optical properties are governed by the linear dielectric permittivity tensor $\hat{\varepsilon }$. In biaxial crystal systems, the real part of $\hat{\varepsilon }$ writes as a diagonal matrix in the dielectric frame (X, Y, Z), leading to three principal values. Recently, we reported from measurements and calculations that the imaginary part of $\hat{\varepsilon }$ is not diagonal in such frame, when dealing with biaxial crystals from the monoclinic crystal system. We investigated the absorption at 812 nm and the emission of luminescence at 1061 nm in the YCOB:Nd crystal belonging to the C_m_ monoclinic symmetry orientation class, where m stands for a mirror plane perpendicular to the Y-axis [3]. We wrote new analytical expressions able to interpolate our measurements in the XZ principal plane of YCOB:Nd, by introducing non-zero extra-diagonal elements in the imaginary part of $\hat{\varepsilon }$. Moreover, by numerically solving the propagation wave equation, we were also able to model the angular distribution of the absorption coefficient at 812 nm of YCOB:Nd that we had measured for directions of propagation out of the principle dielectric planes. Thus, we confirmed that absorption, luminescence and more generally linear spectroscopic properties, are depicted by a double layer-surface in biaxial crystal systems [2,3]. However, to the best of our knowledge, we were the first to claim that these two layers intersect themselves, leading to polarization-independent spectroscopic properties along the associated continuous intersection loci: such affirmation from numerical calculations has been corroborated experimentally [4].

The present work provides a generalization of our previous studies performed in the monoclinic Nd:YCOB crystal. By numerically solving the wave propagation equation in the weak-absorption approximation [2], we depict the angular distribution of luminescence and absorption properties in biaxial crystal systems, since such distribution is directly driven by that of the imaginary part of the optical index $\hat{n}$. As far as we know, we report for the first time the complete three-dimensional angular distribution of the imaginary part of the optical index $\hat{n}$ in biaxial media, leading to an original double-layer surface with topology and symmetry properties that clearly differ from the well-known refractive index surface. Moreover, we point out a nontrivial behavior of angular distributions of luminescence and absorption properties in biaxial media since the two related layers intersect themselves, leading to so-called *layer-inversion zones*. The influence of the biaxial crystal system is studied by successively considering orthorhombic, monoclinic and triclinic systems.

## 2. Wave Propagation Equation

The description of linear optical properties in anisotropic media requires the wave propagation equation to be solved, *i.e.,* the Maxell equation for the considered light-matter interaction. This equation is [2,3,4]:

(1) $${\hat{n}}^{2}\left(\theta ,\varphi \right)\left(\vec{u}(\theta ,\varphi )\times \vec{u}(\theta ,\varphi )\times \vec{E}\right)+\hat{\varepsilon }{\varepsilon_{0}}^{-1}\vec{E}=0$$

where $\vec{E}$ is the propagating electric field, $\vec{u}(\theta ,\varphi )$ is the unit wave vector along the wave propagation direction described by the spherical angular coordinates (θ,φ), × is the vectorial product, $\varepsilon_{0}$ is the vacuum dielectric permittivity, $\hat{\varepsilon }$ is the linear dielectric permittivity tensor that drives both linear propagation and linear spectroscopic properties, and the complex scalar $\hat{n}(\theta ,\varphi )$ is the optical index along the (θ,φ) propagation direction.

The optical index, $\hat{n}(\theta ,\varphi )$, can be written as $\hat{n}(\theta ,\varphi )=n(\theta ,\varphi )+jn\prime (\theta ,\varphi )$, where n(θ,φ) and n′(θ,φ) are the real and imaginary parts, respectively. As well-known, linear spectroscopic properties are directly proportional to n′(θ,φ), so that their angular distribution is highly driven by that of n′(θ,φ). Therefore, the angular distribution of luminescence and absorption properties are directly depicted from calculating the imaginary part of the optical index $\hat{n}$, *i.e.,* the solution of the wave propagation equation for any direction of propagation. Such calculations are performed in the dielectric frame (X, Y, Z), also called the optical frame, which is classically used to depict the index surface [2].

In biaxial media, Equation (1) is solved in the dielectric frame (X, Y, Z), where the second-rank polar tensor that describes the linear dielectric permittivity tensor writes $\hat{\varepsilon }=\varepsilon +j\varepsilon \prime$, leading to [3,4]:

(2) $$\hat{\varepsilon }=\left[\begin{array}{ccc} \varepsilon_{xx} & 0 & 0\\ 0 & \varepsilon_{yy} & 0\\ 0 & 0 & \varepsilon_{zz} \end{array}\right]+j\left[\begin{array}{ccc} {\varepsilon \prime }_{xx} & {\varepsilon \prime }_{xy} & {\varepsilon \prime }_{xz}\\ {\varepsilon \prime }_{yx} & {\varepsilon \prime }_{yy} & {\varepsilon \prime }_{yz}\\ {\varepsilon \prime }_{zx} & {\varepsilon \prime }_{zy} & {\varepsilon \prime }_{zz} \end{array}\right]$$

where *ε_xx_,* *ε_yy_* and *ε_zz_* are the three principal values of the real-part tensor ε, which is systematically diagonal in the dielectric frame of any biaxial crystal system. *ε’_xx_,* *ε’_yy_* and *ε’_zz_* are the diagonal values of the imaginary-part tensor ε′: these elements are the only non-zero elements of ε′, when dealing with orthorhombic crystals. In monoclinic crystals, ε’ also has two non-zero extra-diagonal coefficients that are written *ε’_xz_* = *ε’_zx_* in the case of symmetry orientation classes equivalent to that of YCOB:Nd [3,4]. In the case of triclinic crystals, the nine coefficients of the imaginary part of the dielectric permittivity, *ε’_ij_* with i, j = x, y or z, all show non-zero values, satisfying the relation ${\varepsilon \prime }_{ij}={\varepsilon \prime }_{ji}$ [2,6].

Then, the projection of Equation (1) along the three principal axes of the dielectric frame (X, Y, Z) leads to the following complex linear system of three coupled equations:

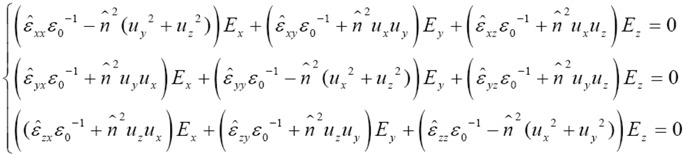
(3)
where (*E_x_, E_y_, E_z_*) and (*u_x_, u_y_, u_z_*) are the Cartesian coordinates of the electric field $\vec{E}$ and of the unit wave vector $\vec{u}$ in the dielectric frame (X, Y, Z), with $u_{x}=\text{sin}(\theta )\text{cos}(\varphi )$*,* $u_{y}=\text{sin}(\theta )\text{sin}(\varphi )$ and $u_{z}=\text{cos}(\theta )$ depending on the spherical angular coordinates (θ,φ).

## 3. Numerical Calculation of the Angular Distribution of Luminescence or Absorption Properties

We performed the numerical resolution of the complex linear system of coupled equations (3) by numerically solving its determinant equal to zero for any given wave direction of propagation: ${\hat{n}}^{2}(\theta ,\varphi )$ is the unknown parameter that remains to be determined for given values of the ${\hat{\varepsilon }}_{ij}$ elements in the considered biaxial medium. This leads to two complex solutions, ${{\hat{n}}^{2}}_{+}(\theta ,\varphi )$ and ${{\hat{n}}^{2}}_{-}(\theta ,\varphi )$, corresponding to the two eigenmodes of polarization ${\vec{E}}_{+}\left(\theta ,\varphi \right)$ and ${\vec{E}}_{-}\left(\theta ,\varphi \right)$, where (θ,φ) are the spherical coordinates of the wave vector. Since refractive indices are strictly positive real numbers in classical media, solutions ${\hat{n}}_{+}(\theta ,\varphi )$ and ${\hat{n}}_{-}(\theta ,\varphi )$ with positive real parts are the only ones to be considered. Real part solutions are $n_{+}(\theta ,\varphi )=\text{Re}\left({\hat{n}}_{+}(\theta ,\varphi )\right)$ and $n_{-}(\theta ,\varphi )=\text{Re}\left({\hat{n}}_{-}(\theta ,\varphi )\right)$, while imaginary ones are ${n\prime }_{+}(\theta ,\varphi )=\text{Im}\left({\hat{n}}_{+}(\theta ,\varphi )\right)$ and ${n\prime }_{-}(\theta ,\varphi )=\text{Im}\left({\hat{n}}_{-}(\theta ,\varphi )\right)$. Note that we use the classical convention $n_{+}(\theta ,\varphi )\geq n_{-}(\theta ,\varphi )$ that only applies to the refractive indices, since no *a priori* relation of order can strictly be imposed to ${n\prime }_{+}(\theta ,\varphi )$ and ${n\prime }_{-}(\theta ,\varphi )$, as further discussed in this paper.

**Figure 1:**
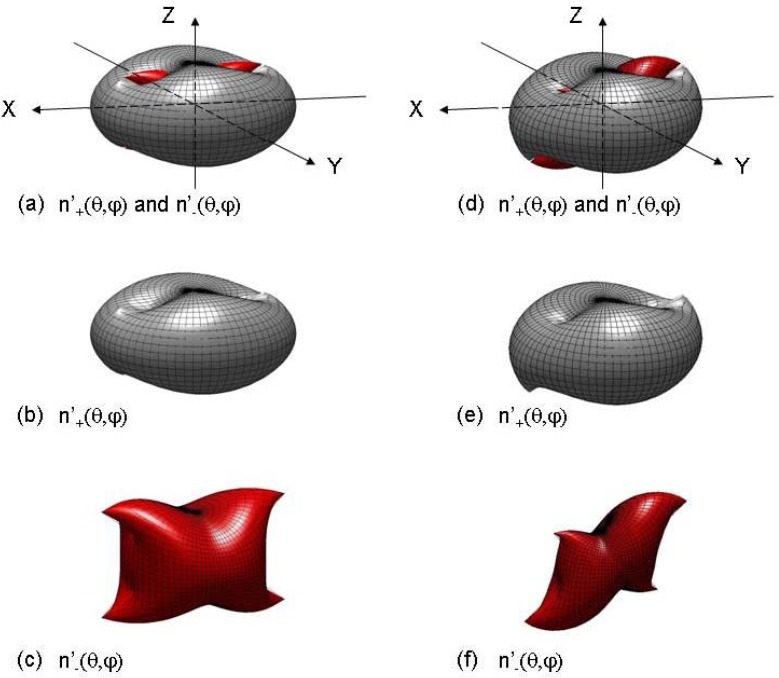
Numerical calculations of angular distributions of luminescence or absorption properties in the dielectric frame (X, Y, Z) of biaxial crystals from the orthorhombic {(a)-(b)-(c)} and monoclinic {(d)-(e)-(f)} crystal systems; (a)-(d) double-layer surface composed of ${n\prime }_{+}(\theta ,\varphi )$ and ${n\prime }_{-}(\theta ,\varphi )$, showing remarkable layer-inversion zones; (b)-(e) and (c)-(f) single layers related to ${n\prime }_{+}(\theta ,\varphi )$ and ${n\prime }_{-}(\theta ,\varphi )$, respectively.

Figure 1 shows ${n\prime }_{+}(\theta ,\varphi )$ and ${n\prime }_{-}(\theta ,\varphi )$ in the dielectric frame (X, Y, Z), describing the angular distribution of luminescence or absorption properties of biaxial crystals from the orthorhombic and monoclinic crystal systems. The case of triclinic crystals is deliberately not depicted here, since no luminescent triclinic material has even been reported yet. Theoretical curves of Figure 1 have been calculated with reasonable values of ${\hat{\varepsilon }}_{ij}$ elements that verify the usual weak spectroscopic properties approximation, *i.e.,* ${\varepsilon \prime }_{ij}<<\varepsilon_{ii}$. In the present work, ${\varepsilon \prime }_{ij}$ values are chosen to be five orders of magnitude smaller than the $\varepsilon_{ii}$ values. Figures 1(a), (b) and (c) depict an arbitrary orthorhombic medium where: *ε_xx_* *ε_0_^-1^* = 2.71, *ε_yy_* *ε_0_^-1^* = 2.89, and *ε_zz_* *ε_0_^-1^* = 2.95 for the real part; *ε’_xx_* *ε_0_^-1^* = 2.20×10^-5^, *ε’_yy_* *ε_0_^-1^* = 3.40×10^-5^, *ε’_xx_* *ε_0_^-1^* = 6.81×10^-5^, and *ε’_xz_* *ε_0_^-1^* = *ε’_xy_* *ε_0_^-1^* = *ε’_yz_* *ε_0_^-1^* = 0 for the imaginary part. On the other hand, Figures 1(d), (e) and (f) illustrate an arbitrary monoclinic medium, with the same parameters to those chosen for the orthorhombic one, except that *ε’_xz_* *ε_0_^-1^* = 2.25×10^-5^. Note that these arbitrarily chosen values are realistic since they are similar to real-life parameters of luminescence properties related to the resonant transition ^4^F_3/2_ ➔ ^4^I_11/2_ at 1061 nm of the Nd^3+^ rare-earth ion in the monoclinic YCOB crystal matrix, which were recently determined by fitting our experimental data of the angular distribution of fluorescence cross-section in Nd:YCOB [3].

Figures 1(a) and (d) show a double-layer surface corresponding to ${n\prime }_{+}(\theta ,\varphi )$ and ${n\prime }_{-}(\theta ,\varphi )$, respectively, for orthorhombic and monoclinic crystals. These surfaces are new mathematical objects that depict the original behavior of the angular distribution of luminescence, but also absorption properties in low-symmetry biaxial media. It is remarkable to observe embedded-layers angular distributions showing topology and symmetry properties that depend on the biaxial crystal system, whereas it is not the case for the well-known refractive index layers $n_{+}(\theta ,\varphi )$ and $n_{-}(\theta ,\varphi )$. The double-layer index surface is the same for all biaxial media, *i.e.,* from orthorhombic, monoclinic and triclinic crystal systems.

Furthermore, our simulations show for the first time that layers ${n\prime }_{+}(\theta ,\varphi )$ and ${n\prime }_{-}(\theta ,\varphi )$ always intersect themselves along continuous loci, as shown in Figures 1(a) and (d). It is emphasized in Figures 1(b), (c), (e) and (f), by independently showing the topologies of layers ${n\prime }_{+}(\theta ,\varphi )$ and ${n\prime }_{-}(\theta ,\varphi )$. Note that such intersection loci are specific to layers ${n\prime }_{+}(\theta ,\varphi )$ and ${n\prime }_{-}(\theta ,\varphi )$. In fact, the index surface, described by $n_{+}(\theta ,\varphi )$ and $n_{-}(\theta ,\varphi )$ layers, shows a double-layer surface with no intersection, but four punctual contacts associated the four ombilics that are located in the XZ plane with the convention *ε_xx_* *ε_0_^-1^* = n_x_ < *ε_yy_* *ε_0_^-1^* = n_y_ < *ε_zz_* *ε_0_^-1^* = n_z_ [2].

Consequently, Figures 1(a) and (d) depict specific directions of propagation where the red layer ${n\prime }_{-}(\theta ,\varphi )$ is observed out of the grey layer ${n\prime }_{-}(\theta ,\varphi )$. We introduce the label of *layer-inversion zones* for these directions, since they correspond to propagation directions that verify ${n\prime }_{+}(\theta ,\varphi )\leq {n\prime }_{-}(\theta ,\varphi )$ with $n_{+}(\theta ,\varphi )\geq n_{-}(\theta ,\varphi )$, whereas out of these layer-inversion zones, one observes ${n\prime }_{+}(\theta ,\varphi )\geq {n\prime }_{-}(\theta ,\varphi )$ with $n_{+}(\theta ,\varphi )\geq n_{-}(\theta ,\varphi )$. The existence of layer-inversion zones implies a crucial consequence when dealing with luminescence or absorption efficiencies: out of these zones, the larger luminescence cross-section (or the larger absorption coefficient) is related to the ${\vec{E}}_{+}\left(\theta ,\varphi \right)$ polarization eigenmode, whereas the smaller luminescence cross-section (or the smaller absorption coefficient) is related to the ${\vec{E}}_{-}\left(\theta ,\varphi \right)$ polarization eigenmode; on the contrary, inside these zones, the larger luminescence cross-section (or the larger absorption coefficient) is now related to the ${\vec{E}}_{-}\left(\theta ,\varphi \right)$ polarization eigenmode, whereas the smaller luminescence cross-section (or the smaller absorption coefficient) is related to the ${\vec{E}}_{+}\left(\theta ,\varphi \right)$ polarization eigenmode. Therefore, the knowledge of the layer-inversion zones is absolutely necessary to properly select the relevant polarization eigenmode to optimize the studied spectroscopic property. Figures 1(a) and (d) show that there are four layer-inversion zones for the chosen parameters. By propagation inversion invariance for opposite directions of propagation, these layer-inversion zones are identical two by two. Moreover, the limits of inversion zones correspond to continuous intersection loci, defined by the condition ${n\prime }_{-}(\theta ,\varphi )={n\prime }_{+}(\theta ,\varphi )$. When used along one given direction of propagation of these intersection loci, biaxial crystals show polarization-independent spectroscopic properties, as experimentally corroborated for luminescence emission at 1061 nm in the biaxial YCOB:Nd crystal from the monoclinic crystal class C_m_. Note that such behavior also occurs for absorption properties, as experimentally measured for the ^4^I_9/2_ ➔ (^4^F_3/2_ + ^2^H_9/2_) absorption transition at 812 nm of the Nd^3+^ that we studied in the same YCOB:Nd crystal. The potential interest of these directions is for example to cope with the thermal load in high-power laser pumping, in order to tend to limit the induced mechanical stress on a luminescent material, depending on the symmetry of dilation tensor [3,4].

## 4. Topology and Symmetry Properties of the Inversion Zones

**Figure 2:**
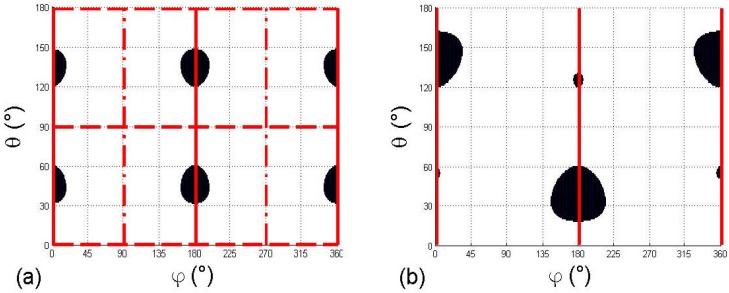
Black zones showing spherical angles coordinates of inversion zones where ${n\prime }_{+}(\theta ,\varphi )\leq {n\prime }_{-}(\theta ,\varphi )$ from the angular distribution depicted in Figure 1. Figures 2(a) and 2(b): Inversion zones related to orthorhombic and monoclinic cases of Figures 1(a) and 1(b), Respectively. Red full lines stand for XZ plane (ϕ = 0°, 180°, 360°), red long-dashed lines for XY plane (θ = 0°, 90°, 180°), and red dotted-dashed lines for YZ plane (ϕ = 90°, 270°) when these dielectric planes are symmetry mirrors for luminescence or absorption properties.

Figure 1 has revealed the existence of layer-inversion zones where the relation of order ${n\prime }_{+}(\theta ,\varphi )\leq {n\prime }_{-}(\theta ,\varphi )$ is fulfilled. So as to provide a better understanding of the related topology and symmetry properties, the locations of the inversion zones from Figure 1 are depicted in a more convenient representation in Figure 2, by showing them with darken zones in the dielectric frame, as a function of the spherical coordinates (θ,φ).

Figure 2(a) provides the inversion zones corresponding to the angular distribution in orthorhombic crystal systems, related to Figures 1(a)−(b)−(c). Since all extra-diagonal elements are null, the three principal planes of the dielectric frame are mirror symmetry planes for the luminescence and absorption angular distribution, as well as it is the case for the refractive index surface. Then it is of importance to note that refractive index, luminescence and absorption angular distributions present the same symmetry elements for orthorhombic biaxial media, even if their own topologies clearly differ. These symmetry mirrors are marked out as vertical red full lines for the XZ principal plane (for ϕ = 0°, 180°, and 360°), as horizontal red long-dashed lines for the XY principal plane (for θ = 90° and 270°) and as horizontal red dotted-dashed lines for YZ principal planes (ϕ = 90° and 270°). Therefore, we observe in Figure 2(a) four identical inversion zones from the angular distributions of luminescence or absorption properties in orthorhombic biaxial systems.

Figure 2(b) illustrates the inversion zones associated to the angular distribution of luminescence or absorption properties in monoclinic crystal systems, from Figures 1(d)-(e)-(f). In this case, some extra-diagonal elements are non-zero, namely *ε’_xz_* = *ε’_zx_*, for the monoclinic symmetry orientation class ${□}_{m}$, when Y-axis is perpendicular to the crystallographic mirror [3,4]. Therefore, the XZ principal plane is the only remaining mirror for the luminescence or absorption angular distribution, and thus for the related inversion zones too. In Figure 2, this mirror is marked out as vertical red full lines (for ϕ = 0°, 180°, and 360°). It leads to four inversion zones being only identical two by two by propagation inversion invariance for opposite directions of propagation.

Finally, in the case of triclinic biaxial crystals, no symmetry mirror is expected in the angular distribution of luminescence and absorption properties.

**Figure 3:**
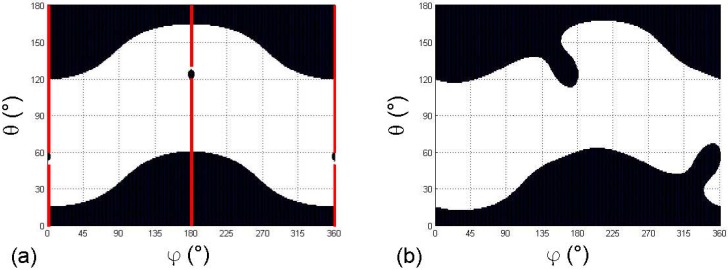
Black area showing spherical angles coordinates of inversion zones. (a) Inversion zones related to the absorption angular distribution at 812 nm in the real-life monoclinic YCOB:Nd crystal. Red full lines stand for XZ plane (ϕ = 0°, 180°, 360°) when this dielectric plane is a symmetry mirror. (b) Possible inversion zones for a theoretical triclinic crystal.

Figure 3 illustrates the potential richness of topology and symmetry properties of inversion zones. It shows that, depending on the relative weight of the chosen parameters ${\varepsilon \prime }_{ij}$, the topology of inversion zones can dramatically change from four to two existing loci.

By using values of ${\varepsilon \prime }_{ij}$ elements that we had experimentally determined [4], Figure 3(a) provides the inversion zones corresponding to the real-life angular distribution of the absorption coefficient at 812 nm of the ^4^I_9/2_ ➔ (^4^F_3/2_ + ^2^H_9/2_) transition of the Nd^3+^ ion measured in the monoclinic luminescent YCOB:Nd crystal: it shows that inversion zones have the same symmetry properties as those of the theoretical angular distribution depicted in Figure 2(a) . It is not surprising since, in both case, we are dealing with linear spectroscopic properties, for the same considered monoclinic biaxial systems belonging to the same symmetry orientation class. However, it also shows that, depending on the chosen sets of parameters ${\varepsilon \prime }_{ij}$, inversion zone topology can change, as seen between Figure 2(b) and 3(a). It is of interest to note that only the measurement of the large inversion zones of Figure 3(a) has been reported by Joly *et al.* [4]. Experimental observation of the smaller inversion zones was never reported up to now, and thus remains challenging from an experimental point of view for both methodological and metrological aspects in luminescent biaxial materials.

Figure 3(b) is plotted with reasonable non-null values for the elements ${\varepsilon \prime }_{ij}$ for a triclinic crystal system. Depending on the relative weight of considered parameters ${\varepsilon \prime }_{ij}$, the topology of triclinic inversion zones can be similar to that of Figures 2(a) and (b), or that of Figure 3(a), showing four inversion zones. Figure 3(b) reveals another possible topology that leads to the merging of inversion zones, finally resulting in only two distinct inversion zones. Since no triclinic luminescent material has been reported up to now, no exhaustive description of all the possible inversion zone topologies is under consideration in the present paper: the goal is here to shine the light on the general specific aspects of the angular distribution related to luminescence or absorption properties in biaxial crystals.

## 5. Conclusion

This theoretical study provides the first complete simulations of the angular distribution of luminescence and absorption properties in biaxial media, which is directly driven by that of the imaginary part of optical indices. While orthorhombic, monoclinic and triclinic crystal systems present the same topology and symmetry properties for the refractive index surface, we show that these three biaxial crystal systems provide distinct imaginary index angular distributions, with distinct topologies and symmetries. We also numerically demonstrate the existence of continuous intersection loci and layer-inversion zones. These zones show a rich diversity of topologies and symmetries, depending on the considered biaxial crystal system. Their complete experimental observation remains challenging, and their knowledge is necessary to properly select the relevant polarization eigenmode to optimize the studied spectroscopic property. Furthermore, their borders provide interesting directions of propagation since they correspond to polarization-independent luminescence and absorption properties.

These general results bring a new fundamental knowledge and understanding of luminescence and absorption properties in all biaxial crystal optics, by demonstrating that the selection of the appropriate polarization eigenmode is not trivial so as to optimize the spectroscopic behavior of low-symmetry media. Therefore, these results bring the keys to fully exploit the potential of linear spectroscopic properties in biaxial innovating luminescent materials [4] as well as of that of self-doubling crystals with both luminescent and nonlinear optical properties [5], fluorescent nano-crystals [8], materials with intrinsic electric conductivity [2] or with laser-induced photoconductivity related to scintillation mechanisms [9]. Finally, it also applies for the interpretation of frequency-degenerate nonlinear light scattering in Sn_2_P_2_O_6_ [10].

## References

[1] Mougel F., Dardenne K., Aka G., Kahn-Harari A., Vivien D. (1999). Ytterbium-doped GdCOB: An efficient infrared laser and self-frequency doubling crystal. J. Opt. Soc. Am. B.

[2] Born M., Wolf E. (1999). Optics of Crystals. Principles of Optics.

[3] Petit Y., Boulanger B., Segonds P., Félix C., Ménaert B., Zaccaro J., Aka G. (2008). Absorption and fluorescence anisotropies of monoclinic crystals: The case of Nd:YCOB. Opt. Express.

[4] Joly S., Petit Y., Boulanger B., Segonds P., Félix C., Ménaert B., Aka G. (2009). Singular topology of optical absorption in biaxial crystals. Opt. Express.

[5] Segonds P., Joly S., Boulanger B., Petit Y., Ménaert B., Aka G. (2009). Laser and self-doubling properties of Nd:YCOB crystal cut as a sphere and inserted in a cavity. J. Opt. Soc. Am. B.

[6] Nye J.F. (1985). Physical Properties of Crystals: Their Representation by Tensors and Matrices.

[7] Landau L., Lifchitz E. (1967). Electromagnetic Waves in Anisotropic Media. Theory of Elasticity.

[8] Vacha M., Kotaki M. (2003). Three-dimensional orientation of single molecules observed by far- and near-field fluorescence microscopy. J. Chem. Phys..

[9] Joubert M.-F., Kazanskii S.A., Guyot Y., Gâcon J.-C., Pédrini C. (2004). Microwave study of photoconductivity induced by laser pulses in rare-earth-doped dielectric crystals. Phys. Rev. B.

[10] Shumelyuk A., Volkov A., Selinger A., Imlau M., Odoulov S. (2008). Frequency-degenerate nonlinear light scattering in low-symmetry crystals. Opt. Lett..

